# Digital PCR for Genotype Quantification: A Case Study in a Pasta Production Chain

**DOI:** 10.3390/biology10050419

**Published:** 2021-05-09

**Authors:** Caterina Morcia, Valeria Terzi, Roberta Ghizzoni, Chiara Vaiuso, Chiara Delogu, Lorella Andreani, Andrea Venturini, Paola Carnevali, Pier Paolo Pompa, Giorgio Tumino

**Affiliations:** 1 Consiglio per la ricerca in agricoltura e l’analisi dell’economia agraria-Centro di Ricerca Genomica e Bioinformatica (CREA-GB), Via San Protaso 302, 29017 Fiorenzuola d’Arda, Italy; caterina.morcia@crea.gov.it (C.M.); roberta.ghizzoni@crea.gov.it (R.G.); chiara.vaiuso97@gmail.com (C.V.); 2 Consiglio per la ricerca in agricoltura e l’analisi dell’economia agraria-Centro di Ricerca Difesa e Certificazione (CREA-DC), Via Emilia km 307, 26838 Tavazzano, Italy; chiara.delogu@crea.gov.it (C.D.); lorella.andreani@crea.gov.it (L.A.); andrea.venturini@crea.gov.it (A.V.); 3 Barilla S.p.A., Via Mantova 166, I-43122 Parma, Italy; paola.carnevali@barilla.com; 4 Nanobiointeractions & Nanodiagnostics, Istituto Italiano di Tecnologia, Via Morego 30, 16163 Genova, Italy; pierpaolo.pompa@iit.it; 5 Plant Breeding, Wageningen University Research, Droevendaalsesteeg 1, 6708PB Wageningen, The Netherlands; giorgio.tumino@wur.nl

**Keywords:** digital PCR (dPCR), simple sequence repeats (SSRs), genotype-by-sequencing (GBS), varietal confirmation, molecular traceability, durum wheat, pasta, quantification of variety, private allele, allelic discrimination

## Abstract

**Simple Summary:**

Digital polymerase chain reaction (dPCR) is a breakthrough technology able to provide an absolute quantification of the target sequence through the compartmentalization of the sample and independent amplifications of the numerous separate compartments. Such technology has recently found several applications in plant science; however, to the best of our knowledge, it has never been applied until now for the detection and quantification of a specific plant variety along a production chain. As proof of concept, a dPCR assay targeted to the quantification of a durum wheat variety routinely used in an Italian premium pasta production chain has been developed.

**Abstract:**

Digital polymerase chain reaction (dPCR) is a breakthrough technology based on the partitioning of the analytical sample and detection of individual end-point amplifications into the separate compartments. Among the numerous applications of this technology, its suitability in mutation detection is relevant and characterized by unprecedented levels of precision. The actual applicability of this analytical technique to quantify the presence of a specific plant genotype, in both raw materials and transformed products, by exploiting a point polymorphism has been evaluated. As proof of concept, an Italian premium pasta production chain was considered and a dPCR assay based on a durum wheat target variety private point mutation was designed and evaluated in supply-chain samples. From the results obtained, the assay can be applied to confirm the presence of a target variety and to quantify it in raw materials and transformed products, such as commercial grain lots and pasta. The performance, costs, and applicability of the assay has been compared to analytical alternatives, namely simple sequence repeats (SSRs) and genotype-by-sequencing based on Diversity Arrays Technology sequencing (DArTseq^TM^).

## 1. Introduction

Traceability is currently considered an important issue for food supply chains in defense of different characteristics of a product, including quality and safety, healthiness, origin of production, and sustainability, among others. Several protocols, devices, and sensors have been developed supporting food traceability to answer to legal requirements and/or voluntary regulations and certifications, as reviewed by Espineira and Santaclara [1]. The traceability is therefore a complex issue, with peculiarities for both food and feed production chains.

This study focuses on a particular traceability request for high-quality pasta production. Italian legislation requires pasta to be produced exclusively with durum wheat semolina. Soft wheat is considered a contaminant for this product and its accidental presence cannot exceed 3%, as indicated by Law *n*.580 of 1967 [2], and by subsequent Decreto del Presidente della Repubblica (D.P.R.) 187, 9 February 2001 [3] and D.P.R. 41, 5 March 2013 [4]. The pasta supply chain requires that the ingredients have to be checked at the level of the plant species used. The control of pasta production at the plant-species level is therefore a legal requirement.

At the variety level, no legal requirement is requested to check the presence of one or more specific cultivars, unless the label states that specification. However, wide variability exists among durum wheat cultivars from the technological and qualitative points of view [5,6]. Moreover, some cultivars are legacies of the past, linked to traditional uses, including bread and pasta [7]. Currently, the commercial interest in bread and pasta made from a single variety or with a prevalence of one or few specific varieties is growing [8]. Consequently, there is increasing interest in the tracking of specific genotypes along the supply chain, from seeds to grains and transformed products.

To ensure both the various players in the supply chain and consumers of the effective presence of a particular cultivar in the finished products, new approaches to track specific varieties are needed, to better defend and valorize specialty products. Many DNA-based technologies have so far been evaluated for plant-variety protection and registration [9] and for wheat genetic-diversity estimation [10]. Simple sequence repeats (SSRs), array-based genotyping, and genotyping-by-sequencing (GBS) are the most popular techniques to return certain identity of a cultivar. All three families of techniques, in order to limit the costs, are based on a reduction of the genome complexity, and are obtained with very different strategies. The genome complexity reduction in GBS is linked to the restriction enzymes used [11], while in SSR markers and SNPs (single nucleotide polymorphisms), arrays are linked to the starting set of genotypes [12]. SSRs have been evaluated as complementary traits in DUS testing of wheat [13]. Seven SNP arrays that are now available in wheat (Wheat 9K, 15K, 35K, 55K, 90K, 660K, and 820K SNP array) have been widely used mainly to detect trait-related genetic loci by QTL mapping and GWAS [14]. Among GBS technologies, Diversity Arrays Technology sequencing (DArTseq^TM^), which starts from a smart reduction of the complexity of the genome to produce both sequence data and SNP markers, was recently applied to a diversity analysis of 80,000 wheat accessions by Sansaloni et al. [15].

The three technologies (SSRs, SNP array, DArTseq) are undoubtedly useful for genetic diversity-based studies and for varietal fingerprinting; however, the last two in particular seem too complex and time-consuming to be used in a production chain to track one specific genotype.

Digital PCR is a breakthrough technology able to provide an absolute quantification of the target sequence through the compartmentalization of the sample and independent amplifications of the numerous separate compartments. Such technology has recently found several applications in plant science, as reviewed by Morcia et al. [16]. However, to the best of our knowledge, it has never been applied until now for the detection and quantification of a variety along a production chain.

As proof of concept, a dPCR assay targeted to the quantification of a wheat variety routinely used in Italian premium pasta production chain has been developed. The performance, costs, and applicability of the assay have been evaluated and compared with other analytical technologies.

## 2. Materials and Methods

### 2.1. Study Workflow

The workflow of the study is summarized below:

1. Establishment of a working collection of 28 durum wheat varieties, selected from among the most extensively cultivated in Italian environments [17];
2. Selection of a durum wheat target variety (TV), whose name cannot be reported in accordance with the protection requirements of sensitive industrial data;
3. Genotype-by-sequencing through DArTseq analysis of the DNAs extracted from certified seeds of TV and of all the cultivars, included in the working collection, and establishment of a SNP database;
4. SNP screening directed to the identification of a private allele of the target variety;
5. Development of a chip digital PCR assay designed for such private allele to identify and quantify the target variety;
6. Evaluation of the applicability of the dPCR assay on reference grain, flour, and pasta samples;
7. Digital PCR analysis of five commercial grain samples;
8. DArTseq analysis of five commercial grain samples;
9. SSR analysis of five commercial grain samples;
10. Comparison among the fingerprinting methods.

### 2.2. Materials

Table 1 summarizes the seed, grain, flour, and pasta samples used in this work.

#### 2.2.1. Seed Samples

Certified seeds of the durum wheat working collection (varieties: Achille, Antalis, Anvergur, Aureo, Babylone, Bronte, Claudio, Core, Iride, Fabulis, Kyle, Kronos, Levante, Maestrale, Marco Aurelio, Miradoux, Monastir, Navigator, Normanno, Odisseo, Orizzonte, Pigreco, Relief, Rusticano, Saragolla, Simeto, Svevo, Tirex) were obtained from the Consiglio per la ricerca in agricoltura e l’analisi dell’economia agraria-Centro di ricerca Difesa e Certificazione (Tavazzano, Italy) seed repository or from the breeders responsible for their maintenance in purity. The seeds were milled using a Cyclotec (FOSS Italia S.r.l., Padova, Italy) at 0.2 mm grid diameter, avoiding any contamination between samples. DNA was extracted from three biological replicates of milled seeds using the DNeasy mericon Food Kit (Qiagen, Milan, Italy), according to manufacturer’s instructions. The evaluation of quality and quantity of the extracted DNA was performed using a Qubit™ fluorometer in combination with the Qubit™ dsDNA BR Assay kit (Invitrogen by Thermo Fisher Scientific, Monza, Italy).

#### 2.2.2. Flour Samples

Flours were obtained from the target variety and nontarget variety (Odisseo cultivar) grains using a Cyclotec (FOSS Italia S.r.l., Padova, Italy) mill at 0.2 mm grid diameter, avoiding any contamination between samples. The flour samples reported in Table 1 containing TV percentages ranging from 90 to 20% were obtained by mixing TV and Odisseo flours. After weighing the two wheat flours, the samples containing different percentages of the two cultivars were homogenized for 10 min. DNAs were extracted from flours (2 g) with the DNeasy mericon Food Kit (Qiagen, Milan, Italy), as previously described. The evaluation of quality and quantity of extracted DNA was performed as described above.

#### 2.2.3. Pasta Samples

Pasta samples were obtained by mixing tap water and wheat flours containing the following TV percentages: 100%, 90%, 70%, 50%, and 20%. The samples were dried in oven at 80 °C for 1 h, followed by 3 h at decreasing temperatures. Such a desiccation thermal profile is among those currently used for industrial pasta preparation. DNAs were extracted from two biological replicates of reference pasta using the DNeasy mericon Food Kit (Qiagen, Milan, Italy). The pasta samples were milled with an M20 Universal Mill (IKA^®^-Werke GmbH & Co., Staufen, Germany). Samples (2 g) were extracted with the DNeasy mericon Food Kit (Qiagen, Milan, Italy). The DNA obtained was measured as described in Section 2.2.1.

#### 2.2.4. Commercial Grain Lots

Five commercial grain lots expected to consist of the target variety only were found, and 50 g of bulked grains were sampled from each lot. DNA was extracted from each sublot using the DNeasy mericon Food Kit (Qiagen, Milan, Italy) as already reported. These DNA samples were used for the SSR and dPCR analyses. For the DArTseq analysis, the DNAs were extracted from single seeds using the same method. Each commercial sample was represented for DArTseq analysis by 15 single seeds.

### 2.3. Chip Digital PCR Assay

Genotype-by-sequencing based on DArTseq technology and SNP screening for a TV private allele were performed according to Cibecchini et al. [18]. After screening, the SNP 12876 was selected. It is localized on chromosome 7A of durum wheat, where the reference allele is G and the alternative is T. The T allele is present only in the TV, while the other varieties tested were homozygous G/G. The BLAST of the SNP 12876 flanking sequence on the Svevo durum wheat reference genome highlighted even the presence of a homeologous region on chromosome 7B with high identity. The homeologous chromosome 7B held a nonpolymorphic G/G locus in all the tested varieties.

Primers and MGB probes were designed on the SNP 12876 sequence using the Custom TaqMan^®^ SNP Genotyping Assay procedure (Thermo Fisher Scientific, Monza, Italy), and are available as Assay ID ANXGZMY, Catalog *n*. 4332077 (Thermo Fisher Scientific, Monza, Italy). In the dPCR assay developed, the TV target allele was marked with FAM, whereas the alternative, non-TV allele was marked with VIC.

Chip digital PCR was performed using the QuantStudio^TM^ 3D Digital PCR System (Applied Biosystems by Life Technologies, Monza, Italy). The reaction mixture was prepared in a final volume of 16 µL consisting of 8 µL QuantStudio^TM^ 3D Digital PCR 2X Master Mix, 0.4 µL of Custom TaqMan^®^ SNP Genotyping Assay 40X (Catalogue number 4332077, Applied Biosystems by Life Technologies, Monza, Italy) containing primer and VIC/FAM-MGB probes, 1 µL of DNA (10 ng/µL), and nuclease-free water. In addition, a negative control with nuclease-free water as a template was added. A total volume of 15 µL of reaction mixture was loaded onto the QuantStudio^TM^ 3D Digital PCR chips using the QuantStudio^TM^ 3D Digital chip loader, according to the manufacturer’s protocol. Amplifications were performed in a ProFlex^TM^ 2Xflat PCR System Thermocycler (Applied Biosystems by Life Technologies, Monza, Italy) under the following conditions: 96 °C for 10 min, 47 cycle of 60 °C annealing for 2 min, and 98 °C denaturation for 30 s, followed by 60 °C for 2 min and 10 °C. End-point fluorescence data were collected in a QuantStudio^TM^ 3D Digital PCR Instrument, and the files generated were analyzed using cloud-based platform QuantStudio^TM^ 3D AnalysisSuite dPCR software, version 3.1.6. Each sample was analyzed in triplicate.

### 2.4. SSR Analysis

For the analysis of TV, Odisseo certified seeds, commercial grain lots, and pasta samples, 14 SSR markers were used as described in the *International Rules for Seed Testing 2021* [19] for wheat varieties. For each DNA sample, the amplification was performed in duplicate.

**Figure 1:**
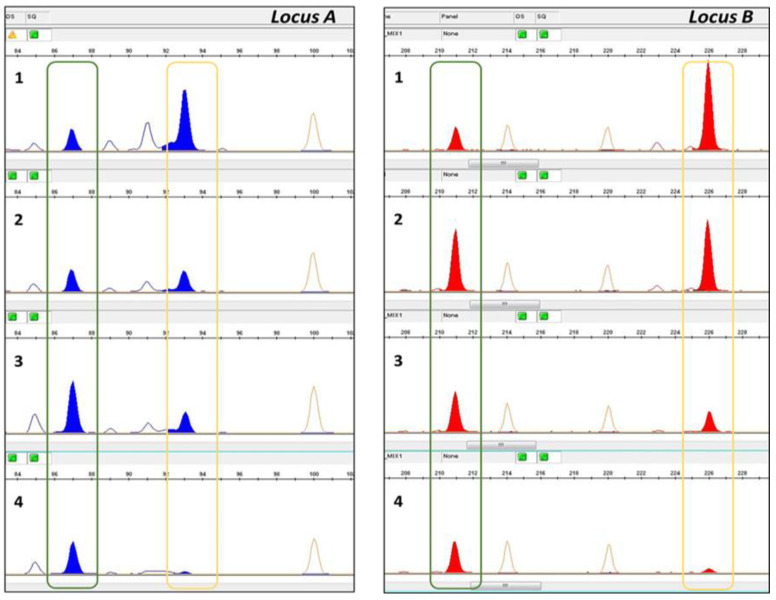
Electropherograms showing amplicons at two polymorphic loci between the TV and Odisseo. The green line highlights the TV alleles, while the yellow line highlights the Odisseo alleles. Plots of Locus A and Locus B from 1 to 4 show the electropherogram obtained from the pasta samples (1: TV 20%–Odisseo 80%, 2: TV 50%–Odisseo 50%; 3: TV 70%–Odisseo 30%; 4: TV 90%–Odisseo 10%).

Each SSR forward primer was labeled with a fluorescent dye on the 5′ end (6-FAM, VIC, HEX, NED, PET) and the 14 SSRs were amplified in two multiplex PCR reactions. PCR amplifications were performed in 10 µL reaction volumes containing 1 µL of 10 ng/µL genomic DNA as a template, 5 µL of 2X Type-it Multiplex PCR Master Mix (Qiagen, Milan, Italy), 1 µL 10x primer mix (2 µM each primer, Thermo Fisher Scientific, Monza, Italy), and 3 µL RNase-free water. The PCR program consisted of initial denaturation for 5 min at 95 °C, followed by 26 cycles of 30 s at 94 °C, 90 s at 57 °C, 30 s at 72 °C, and 1 cycle of 30 min at 72 °C. The PCR products were separated by capillary electrophoresis on the 3500 Genetic Analyzer (Applied Biosystems by Life Technologies, Monza, Italy). For each amplified fragment base pair size, height and peak area were measured by v5 GeneMapper software (Applied Biosystems by Life Technologies, Monza, Italy). The TV was quantified based on the relative quantities of its specific allelic fragment vs. all amplified alleles for the concerned locus, in terms of peak area [20,21]. The estimated percentage was calculated as the average of the peak area value obtained from the polymorphic loci. Figure 1 shows an example of polymorphisms between the TV and Odisseo cultivar and the different peak sizes according to different TV and Odisseo percentages.

### 2.5. DArTseq Analysis

According to point 8 of the study workflow cited in Section 2.1, DNAs extracted from 15 single seeds of five commercial grain samples, together with the DNAs extracted from 15 single seeds sampled from the TV certified seed lot, were sent to Diversity Arrays Technology Pty Ltd. (http://www.diversityarrays.com, accessed on 8 May 2021, Canberra, Australia) for sequencing, and SNP marker identification was done by DArTseq genotyping. The data were curated to include only SNP markers with NA <5% and MAF >5%. The final data set included 6249 SNPs. Euclidean genetic distances were calculated between each pair of samples and further used in clustering analysis (R stats: hclust, method = “average”). The intravarietal genetic distance present in the TV cultivar was calculated on the data obtained from the 15 certified single seeds. Such value was considered as the maximum intravariety distance present in the TV cultivar. Single seeds present in the five commercial samples with higher values of genetic distance were considered different from the TV. The TV percentages in the five commercial samples were then calculated as TV seeds/15 seeds.

## 3. Results

### 3.1. Digital PCR Assay

#### 3.1.1. Specificity

**Figure 2:**
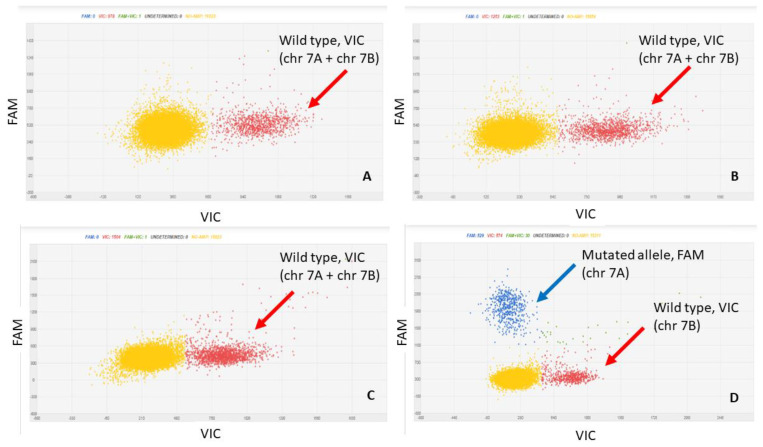
Two-dimensional scatter graphs generated by chip digital PCR (cdPCR) analysis of DNAs from the durum wheat varieties: Miradoux (**A**), Maestrale (**B**), Odisseo (**C**) and target variety (**D**). The G allele is marked by VIC (red cloud), and the T allele by FAM (blue cloud). All the patterns have a yellow cloud due to DNA-empty wells.

The assay was developed with the aim to be a confirmation assay; that is, with the aim of verifying whether the target variety was actually present in the supply chain sample and in what quantities. According to this objective, the assay was built on target variety private alleles; that is, present in only one of the analyzed varieties. As reported in the Materials and Method section, in the dPCR assay, the mutated T allele (only present in target variety) was marked with FAM, whereas the wild type G allele, present in all the varieties, was marked with VIC. The dPCR assay developed was specific for target variety identification and quantification, as verified in DNA samples extracted from certified seeds of the TV cultivar and of the durum wheat varieties of the working collection. Examples of amplification patterns obtained are reported in Figure 2.

The dPCR assay on the 100% target variety sample detected both alleles with equal concentration in terms of copies/µL. The test in fact amplified the SNP target region on chromosome 7A (TT in target variety and GG in the other varieties) and a second locus on the homeologous chromosome 7B (nonpolymorphic, GG in all varieties). Supplementary Figure S1, shows the alignment of the chromosome 7A SNP 12876 sequence to the durum wheat Svevo reference genome. A high-identity region was mapped on homeologous chromosome 7B as well.

#### 3.1.2. Precision, Accuracy, Trueness, and Sensitivity

**Figure 3:**
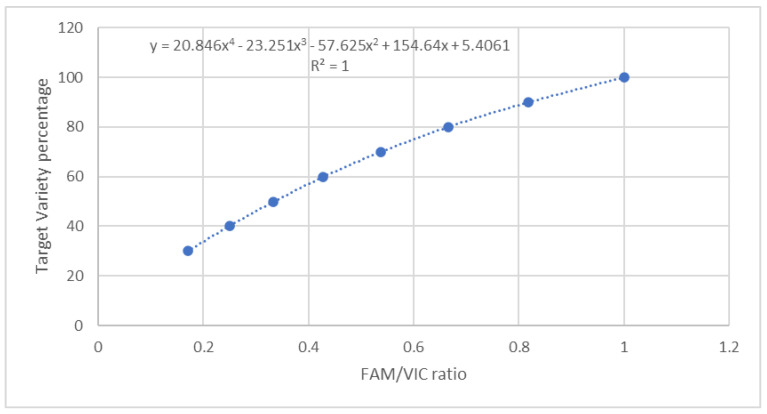
Theoretical correlation between TV percentage and FAM/VIC ratio.

The dPCR assay was applied to TV quantification in the reference flours obtained by mixing TV and non-TV flours in percentages ranging from 100% TV to 0% TV (Table 1). As reported in the Materials and Methods section, the TV target allele was marked with FAM, whereas the alternative allele was marked with VIC. Both FAM and VIC signals were present in equal quantity in the TV pure samples, because TV chromosome 7A carries the T allele, whereas TV chromosome 7B carries the G allele, as already reported. On the contrary, all the other varieties had VIC signals only, because G alleles were present in both chromosomes 7A and 7B. Moreover, in 100% TV samples, the G and T alleles were amplified with very similar efficiency, as demonstrated by the FAM/VIC ratio of 0.98 ± 0.4 experimentally found. Consequently, the curve reported in Figure 3 and the related polynomial function were calculated to correlate the TV percentage present in a theoretical sample and the expected FAM/VIC ratio. Such polynomial function (Figure 3) was experimentally validated in the flour and pasta reference samples reported in Table 1. Table 2 shows the obtained results.

The SD values (Table 2) were <35% for all samples and therefore the precision of the method was acceptable, according to the Codex Alimentarius Commission/Guidelines 74–2010 [22]. The accuracy of the method was evaluated by calculating the absolute and relative errors (Table 2). The trueness of the dPCR assay fit with the GMO analytical guidelines [23] because the estimated percentages over the dynamic range tested were within the ±25% acceptable bias, as recommended. A mean bias of 0.05 ± 0.09 was in fact found between the theoretical and experimentally determined estimated percentages in the flour samples. The sensitivity of the assay was found to be a 0.124% level of contamination. The Pearson’s r between the actual and the experimentally determined percentages was 0.995 for the flour samples.

### 3.2. Digital PCR Assay Validation on Reference Pasta and Comparison with SSR Analysis

Four reference pasta samples, prepared as described in the Material and Methods section, starting from mixed TV + Odisseo flours, were analyzed with the dPCR technique. The results obtained are reported in Table 3. The Pearson’s r between the actual and the experimentally determined percentages in pasta samples was 0.991.

The same four reference pasta samples also were analyzed with the SSR technique. As a preliminary step, the certified seed samples of the TV and Odisseo were genotyped using the 14 SSR markers to obtain the molecular profile. The Odisseo variety and the TV showed two different polymorphic alleles at two SSR loci, considered as “specific marker alleles”. The pasta samples were genotyped and then screened at the two selected SSR marker loci to detect TV and non-TV marker alleles.

In all the pasta samples, it was possible to recognize the alleles of the TV and Odisseo varieties, and then proceed to the detection of the peaks parameters useful for the quantification. The values obtained were repeatable in the different loci and the two replicate samples of each mixture. The mean values were consistent with the actual values of the TV percentage in pasta as reported in Table 3. The Pearson’s r between the actual and the experimentally determined percentages in the pasta samples was 0.998 with SSR analysis.

### 3.3. Digital PCR Assay Application to Commercial Grain Samples and Comparison with SSR and DArTseq Analyses

**Figure 4:**
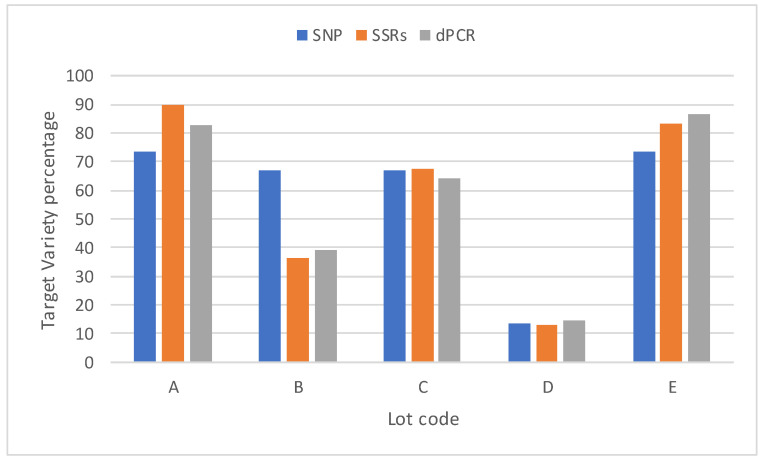
TV percentages found in five grain commercial lots measured with three different approaches (dPCR, SSR, and DArTseq).

The dPCR assay was used to evaluate the TV percentages present in five TV-declared grain commercial lots, but suspected to be contaminated by non-TV varieties. The same samples also were evaluated with SSR and DArTseq approaches, with the same objective; i.e., to evaluate the TV percentages. Figure 4 shows the results obtained with the three different analytical methods.

The Pearson’s r between the TV percentage determined in the commercial lots with dPCR and SSR was 0.991, whereas the Pearson’s r between the TV percentages calculated according to the dPCR and DArTseq analyses were 0.852 and 0.834 for the SSR and DArTseq approaches. In particular, the DArTseq analysis seemed to overestimate the TV percentage in commercial lot B.

## 4. Discussion

A new dPCR-based assay was developed to track a specific genotype. The assay can be applied to confirm the presence of such genotype and to quantify it in raw materials and transformed products. The working hypothesis, i.e., the possibility of exploiting a point polymorphism to confirm or not and quantify the presence of a genotype mixed with others, was fully confirmed. To the best of our knowledge, this is the first example of dPCR application to the quantification of a cultivar obtained after the conventional breeding procedure. Several dPCR assays have in fact been developed to track genetically modified events, but not conventional varieties [16,24]. This last goal raised the level of complexity related to the development of the assay. In fact, in the case of GM events, the target sequence is a priori known, requiring, for the purposes of authorization for cultivation and use, detailed information on the transgenic sequences inserted and on the surrounding genomic areas. On the contrary, in the case of a conventional variety, it is necessary to identify, as the first step of the workflow, one or few private polymorphisms that uniquely identify the target variety. To this purpose, a database of SNP profiles derived from DArT-based genotyping by sequencing characterization was exploited. A panel of durum wheat varieties was selected from among those extensively cultivated in Italy, and therefore at greater risk of being confused or harvested and stored together with the target variety. This therefore confirms the existence of other cultivars with the same sequence polymorphism used for this discrimination assay. However, the chosen polymorphism is effective for an application in the actual supply chain under consideration. Moreover, it can always be accompanied by further markers if such a need arises in the production chain.

From the obtained results, it can be concluded that the dPCR technique demonstrated to be usefully applied for varietal quantification not only in grains and flours, but also in processed products; e.g., in pasta, which can be subjected to high temperatures during drying. The reliability of dPCR for analysis of heat-treated samples has been previously demonstrated, and dPCR proved to be superior to real-time quantitative PCR in testing for genetically engineered events in such heat-treated samples [25].

To compare the dPCR approach with other analytical alternatives, a common set of commercial grain samples was analyzed not only with dPCR, but also with two other techniques, based on SSR and SNP markers.

SSR are consolidated markers in the varietal fingerprinting of many agricultural species and, for some of these, sets of internationally shared SSR markers have been identified. In the case of the *Triticum* species, including durum wheat, the International Seed Testing Association (ISTA) developed and published a standard protocol based on 14 SSRs internationally agreed upon for variety testing and evaluation of seed lots [19]. The 14 SSR markers used showed a high level of polymorphism. Three to six different polymorphic loci were recognized in the commercial grain samples. In lot D, with an estimated TV percentage of 13.2%, 18 different alleles were scored, while the TV and Odisseo were differentiated by two loci. The ISTA protocol is based on a semiperformance approach: in case it is necessary to distinguish very similar varieties, the number of SSR markers can be increased to improve the discrimination power of the protocol. However, the multiplex PCR assay reduces the time and costs of the analysis. The quantification is the result of the qualitative–quantitative evaluation of the total polymorphic alleles obtained. The evaluation can be time-consuming when the nontarget varieties are numerous. In brief, due to the robustness of SSR in varietal fingerprinting, the CE (capillary electrophoresis) quantification method could give results close to those obtained with more innovative technologies.

SNPs are the markers of choice for mapping traits of interest, to assess the level of genetic diversity of a population, to study its structure, or to reconstruct genetic relationships among accessions. SNP markers were used to evaluate the varietal identity in commercial lots using a single-seed analytical procedure. Each commercial batch was represented by 15 seeds, which were genotyped individually. Lower correlations among SNP-based results and dPCR- or SSR-based ones in commercial lots were observed, in comparison with the very high correlations found between dPCR and SSR data for the same samples. This evidence leads to the hypothesis that this method, although able to give a careful description of the genotype, was more sensitive to sampling than the others. For SNP analysis, in fact, 15 seeds were genotyped, whereas for the dPCR and SSR analyses, the DNAs were extracted starting from 50 g of bulked and milled grains, which meant more than 1000 seeds. On the other hand, the SNP-based analysis was the key step for the identification of private alleles needed for dPCR assay development.

## 5. Conclusions

This is the first example of development and application of a dPCR assay aimed at confirming the authenticity of a supply-chain product. The approach was fully satisfactory in terms of precision, accuracy, trueness, sensitivity, and applicability. It can therefore open the way to subsequent applications in various production chains. Moreover, compared to the other reference techniques, it is the only one to have the characteristics close to a DNA barcoding, intended as taxonomic method that uses one short genetic sequence for identification at the species level. The same target genetic sequence can be exploited in fast methods, as reported for instance in [18]. This latter point-of-care (POC) method is user-friendly and fast, yet the dPCR assay is able to provide an accurate quantification. SSR analysis also proved to be effective in quantifying a target variety in raw materials and processed products, with sensitivity close to dPCR. On the contrary, the DArTseq approach, which is positioned at the opposite extreme of the concept of barcoding, cannot be proposed for efficient quantification of varietal components.

By focusing on analytical costs, digital PCR analysis is in the lower price class when compared to other analytical approaches. Table 4 shows the approximate analytical costs related to the characteristics of each assay for the quantification of a specific variety in one single sample. In addition to the methods reported in this work, other approaches that proved to be useful for genotype quantification (reviewed by Madesis et al. [26]) have been considered, such as high-resolution melting (HRM) single-assay platforms, qPCR and SSR, or high-throughput genotyping. The costs were partly deduced from rate tables present on the Italian market and were grouped into classes that included ranges of costs.

Considering a single-sample analysis, the dPCR and SSR peak area evaluations are the cheaper methods, as they do not need to develop a reference curve for quantitation, or a single-seed based analysis. Increasing the number of samples to be analyzed at the same time, the qPCR and HRM-based methods also fall into class A of costs, since the development cost of the reference curve is amortized by the number of analytical samples. On the contrary, all the genotyping methods that need to analyze several sampled seeds to obtain a quantitative result maintain a high cost.

In conclusion, starting from the pilot work developed here, it can be said that the dPCR has a useful role in verifying and confirming the authenticity of agro-food products. This applicability is strengthened by the lower analytical costs and by the reduced analytical times compared to other methods, as well as by its precision in quantitative analyses in comparison with POC approaches.

## Figures and Tables

**Table 1:** The samples used in this work and the techniques used for their analysis.

Sample	dPCR	DArTseq	SSR
Working collection of certified seeds	+	+	+
100% TV flour	+	−	−
90% TV flour	+	−	−
80% TV flour	+	−	−
70% TV flour	+	−	−
60% TV flour	+	−	−
50% TV flour	+	−	−
40% TV flour	+	−	−
30% TV flour	+	−	−
20% TV flour	+	−	−
0% TV flour	+	−	−
Pasta 100% TV	+	+	+
Pasta 90% TV	+	+	+
Pasta 70% TV	+	+	+
Pasta 50% TV	+	+	+
Pasta 20% TV	+	+	+
Grain commercial lots	+	+	+

**Table 2:** Actual TV percentages in comparison with those dPCR experimentally determined (“Mean TV% in flour”) in mixed flour samples, prepared as described in the Materials and Methods section.

Actual TV% in Flour	Mean TV% in Flour	Std Dev	Absolute Error	Relative Error
**100%**	96.6	0	3.4	0.03
**90%**	90.9	0.07	0.95	0.01
**80%**	84.2	0.47	4.2	0.05
**70%**	70.3	0.56	0.3	0.004
**60%**	55.7	2.48	4.25	0.07
**50%**	48.7	1.63	1.25	0.025
**40%**	39.7	2.62	0.25	0.006
**30%**	31.4	1.84	1.4	0.04
**20%**	26.1	0.92	6.15	0.3
**0%**	0	-	-	-

**Table 3:** Actual TV percentages in comparison with those dPCR and SSR experimentally determined in reference pasta samples prepared with mixed TV and non-TV flours.

**Actual TV% in Pasta**	**Mean TV% in Flour (dPCR)**	**Std Dev**	**Absolute Error**	**Relative Error**
90%	88.7	1.34	1.25	0.01
70%	63.4	2.69	6.6	0.09
50%	48.4	2.05	1.55	0.03
20%	26.1	0.92	6.15	0.31
**Actual TV% in Pasta**	**Mean TV% in Flour (SSR)**	**Std Dev**	**Absolute Error**	**Relative Error**
90%	89	0.02	1	0.01
70%	66	0.01	4	0.06
50%	49	0.03	1	0.02
20%	20.5	0.01	0.5	0.025

**Table 4:** Cost evaluation for the quantitative detection of a specific genotype in one wheat grain sample. Class A includes costs up to EUR 100, class B in the range of EUR 100–200, and class C above EUR 200. The need for a reference curve or for single-seed based analyses is reported as + (needed) or − (not needed). qPCR = quantitative PCR; Bar = barcoding; KASP = kompetitive allele specific.

Analytical Technique	Cost Class	Need of Reference Curve	Need of Single-Seed Analysis
Digital PCR	A	−	−
qPCR	B	+	−
Bar-HRM, SSR-HRM, SNP-HRM	B	+	−
SSR-peak area	A	−	−
SSR genotyping	C	−	+
KASPar SNP genotyping	C	−	+
SNP genotyping	C	−	+
GBS genotyping	C	−	+

## Data Availability

Data is contained within the article or supplementary material.

## Supplementary Materials

The following are available online at https://www.mdpi.com/article/10.3390/biology10050419/s1, Figure S1: Alignment of a 630 bp fragment (from 323 to 955 of the SNP12876 sequence) showing 95% identity with a region of chromosome 7B. SNP 12876 is green highlighted.
