# Supplementary material for: Digital PCR for Genotype Quantification: A Case Study in a Pasta Production Chain
Source: Biology (Basel). 2021 May 9;10(5):419. doi: 10.3390/biology10050419 (PMC8151192; doi:10.3390/biology10050419)
Supplement: Supplementary file 1 [file biology-10-00419-s001.zip › biology-1197368-supplementary.pdf]

```
>gnl|Td Svvo|whe Td AB Svevo 7B
          Length = 722970987

 Score = 1019 bits (514), Expect = 0.0
 Identities = 604/633 (95%), Gaps = 2/633 (0%)
 Strand = Plus / Plus


Query: 323       gagctcgtgcgctccctcggccgcttgccgtccgcgaccaccgcgatggtcgacgccacc
                 |||||||| |||||||||||||||||||||||||||||||||||||||||||||||||||
Sbjct: 533407836 gagctcgtccgctccctcggccgcttgccgtccgcgaccaccgcgatggtcgacgccacc


Query: 383       acttcctcaggtatgtggaagctactcaattacagtctacagtacaatcagtcactgcag
                 || ||||||||||||||| ||||||||||||||||||||||||| |||||||| ||||||
Sbjct: 533407896 acctcctcaggtatgtggtagctactcaattacagtctacagtaaaatcagtccctgcag


Query: 443       tactgcactactgcaccattcaggacgctgacgccgacggggtgtgtttgcaggcgaggg
                 ||| ||||||||||||||||||||  ||||| ||||||||  ||||||||||||||||||
Sbjct: 533407956 taccgcactactgcaccattcaggcggctgatgccgacgg--tgtgtttgcaggcgaggg


Query: 503       ggacgggaggtttccgccggagctgagcctggaggccgtgttccggtgcgtgcggatagg
                 ||||||||||||||||||||||||||||||||||||||||||||||||||||||||||||
Sbjct: 533408014 ggacgggaggtttccgccggagctgagcctggaggccgtgttccggtgcgtgcggatagg


Query: 563       gccggtggacgagccggacgcggagttcgcgtaccagacggcggtgagcatcggggggca
                 ||||||||||||||||||||||||||||||||||||||| ||||||||||||||||| ||
Sbjct: 533408074 gccggtggacgagccggacgcggagttcgcgtaccagacagcggtgagcatcgggggcca


Query: 623       cacgttcaaggggatcctgcgggaccatgggccggcggaagaggcggctgggcagctgcc
                 ||||||||||||||||||||| ||||||||||||||||||||||||||||||||||||||
Sbjct: 533408134 cacgttcaaggggatcctgcgcgaccatgggccggcggaagaggcggctgggcagctgcc


Query: 683       gccgtcctcggcggagtaccaccagctgacaggggccgcgagggagggttcatcgccggc
                 |||||| || | |||||||||| |||||||||| |||||||||||||| |||||||||||
Sbjct: 533408194 gccgtcgtcaggggagtaccacgagctgacaggagccgcgagggaggggtcatcgccggc


Query: 743       cgggagcagcgaggcggccggggggcacggagcgacggtggcgacgtccgcggcggtgct
                 ||||||||||||||||||||| |||||||| ||||||||||||||||| |||||||||||
Sbjct: 533408254 cgggagcagcgaggcggccggcgggcacggggcgacggtggcgacgtctgcggcggtgct


Query: 803       catggacccctacccgacgccgatcggcgccttcgcagcaggcacccagttcttccctca
                 ||||||||||||||||||||||||||||||||||||||||||||||||||||||||||||
Sbjct: 533408314 catggacccctacccgacgccgatcggcgccttcgcagcaggcacccagttcttccctca


Query: 863       taaccctagaacctagcttcaattcctgccatgaattcctagcaagtttcatttactgcc
                 |||||||||||||||||||||||||||||||||||||||| |||||||||||||||||||
Sbjct: 533408374 taaccctagaacctagcttcaattcctgccatgaattccttgcaagtttcatttactgcc


Query: 923       aaagatgatggtcgagttgcattgatccaagtc 955
                 |||||||  |  ||||||||||| |||||||||
Sbjct: 533408434 aaagatggcgaccgagttgcatttatccaagtc 533408466
```

Figure S1. Alignment of a 630 bp fragment (from 323 to 955 of the SNP12876 sequence) showing 95% identity with a region of chromosome 7B. SNP 12876 is green highlighted.
